# Combining Precursor and Fragment Information for Improved Detection of Differential Abundance in Data Independent Acquisition*[Fn FN2]

**DOI:** 10.1074/mcp.RA119.001705

**Published:** 2019-12-30

**Authors:** Ting Huang, Roland Bruderer, Jan Muntel, Yue Xuan, Olga Vitek, Lukas Reiter

**Affiliations:** ‡ Northeastern University, Boston MA 02115; § Biognosys, Wagistrasse 21, 8952 Schlieren, Switzerland; ¶ Thermo Fisher Scientific, 28199 Bremen, Germany

**Keywords:** Quantification, Mass Spectrometry, SWATH-MS, Cancer Biomarker(s), Lung Cancer, Label-Free Quantification

## Abstract

In bottom-up, label-free discovery proteomics, biological samples are acquired in a data-dependent (DDA) or data-independent (DIA) manner, with peptide signals recorded in an intact (MS1) and fragmented (MS2) form. While DDA has only the MS1 space for quantification, DIA contains both MS1 and MS2 at high quantitative quality. DIA profiles of complex biological matrices such as tissues or cells can contain quantitative interferences, and the interferences at the MS1 and the MS2 signals are often independent. When comparing biological conditions, the interferences can compromise the detection of differential peptide or protein abundance and lead to false positive or false negative conclusions.

We hypothesized that the combined use of MS1 and MS2 quantitative signals could improve our ability to detect differentially abundant proteins. Therefore, we developed a statistical procedure incorporating both MS1 and MS2 quantitative information of DIA. We benchmarked the performance of the MS1-MS2-combined method to the individual use of MS1 or MS2 in DIA using four previously published controlled mixtures, as well as in two previously unpublished controlled mixtures. In the majority of the comparisons, the combined method outperformed the individual use of MS1 or MS2. This was particularly true for comparisons with low fold changes, few replicates, and situations where MS1 and MS2 were of similar quality. When applied to a previously unpublished investigation of lung cancer, the MS1-MS2-combined method increased the coverage of known activated pathways.

Since recent technological developments continue to increase the quality of MS1 signals (*e.g.* using the BoxCar scan mode for Orbitrap instruments), the combination of the MS1 and MS2 information has a high potential for future statistical analysis of DIA data.

## Abstract (precis)

DIA profiles of complex biological matrices such as tissues can contain quantitative interferences, and the interferences at the MS1 and the MS2 signals are often independent. We developed a statistical procedure incorporating both MS1 and MS2 quantitative information of DIA. We benchmarked the performance of the MS1-MS2-combined method to the individual use of MS1 or MS2 in DIA. In the majority of the comparisons, the combined method outperformed the individual use of MS1 or MS2.

## Graphical Abstract

**Figure:**
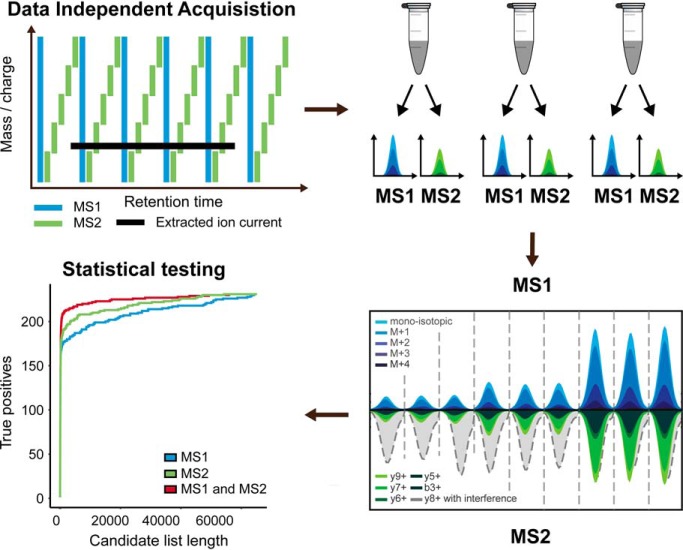

**Highlights**

- Modern DIA methods contain high quality MS1 and MS2.
- We developed a statistical procedure incorporating MS1 and MS2.
- Benchmarking, the combined method outperformed the individual use of MS1 or MS2.

Liquid chromatography-mass spectrometry (LC-MS)^1^ has proven to be a powerful and versatile tool to quantify changes in protein abundance (1). In bottom-up proteomics, proteins are digested into peptides, which are then subjected to mass analysis. Two types of spectra are independently recorded: 1) the intact peptide mass (more precise *m/z*) or MS1 isotope envelope and 2) after fragmenting the peptide, the fragment ion spectrum (MS2). The accurate masses in the MS1 and MS2 spectra are used to identify and/or quantify the peptide. Unfortunately, MS1 and MS2 spectra can contain interferences that distort the quantitative signals. Interferences in MS1 spectra are typically caused by other coeluting peptide precursor isotope envelopes. Interferences in MS2 spectra are typically caused by fragments from coeluting peptides, which occur independently from the interferences in MS1 (2).

The two main applications of LC-MS in discovery-oriented investigations are data-dependent acquisition (DDA) and data-independent acquisition (DIA). In DDA, the MS1 precursor isotope envelope is used to generate extracted ion currents or three-dimensional-peak reconstructions for identification and quantification (3). Additionally, dependent on the MS1 scan, a limited number of peptide precursor peaks are isolated, fragmented, and subjected to secondary mass analysis. These MS2 scans are used for identification but do not contain direct quantitative information. They are not triggered at a defined point in the peptide precursor elution (Fig. 1*A*). In DDA with isobaric labeling, only MS2 information can be used for quantification, *e.g.* reporter ions with ITRAQ or tandem mass tag labels (4, 5) fragment with label remnants for tandem mass tag or easily abstractable sulfoxide-based isobaric-tag (6–8).

**Fig. 1.**
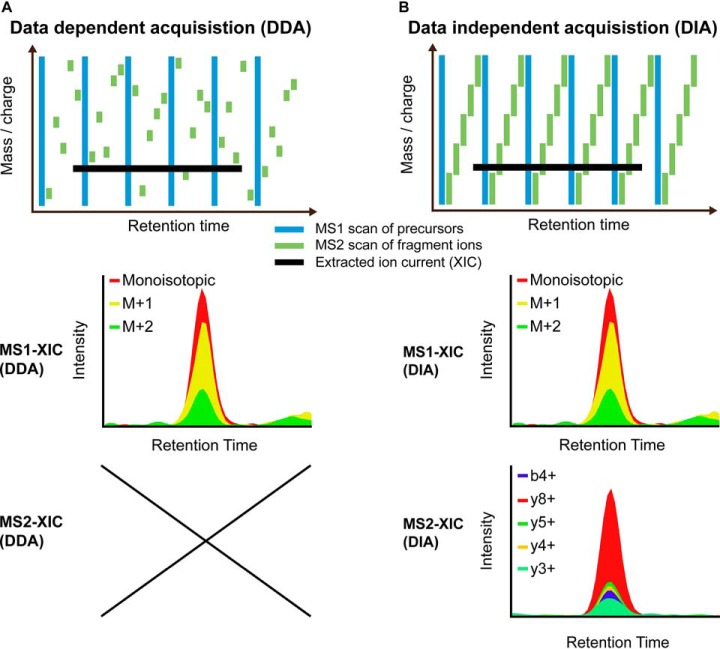
**Quantitative data structure label-free discovery proteomics.** (*A*) Schematic representation of the acquisition layout of data-dependent acquisition methods with regular MS1 scans. The lower panels show the extracted ion current in MS1, which can be used for quantification. (*B*) Schematic representation of the acquisition layout of data-independent acquisition experiment with a regular MS1 and MS2 pattern. The lower panels show the two extracted ion currents, which can be used for quantification.

In contrast, in sequential window acquisition of all theoretical fragment ions (SWATH-type) DIA, MS1 and MS2 data are generated and recorded at a high enough frequency and quality to robustly sample the chromatographic peak (9, 10). A peak group of an extracted ion currents (precursor isotopes or fragments) exists for every peptide precursor in both the MS1 and MS2 space (Fig. 1*B*). In MS1, isotopic variants of an intact peptide precursor are present. In MS2, a precursor is fragmented into multiple fragment ions with isotopic variants. Hence, both quantitative spaces are fundamentally different, and the coeluting interferences do not correlate. Interferences on both levels can be corrected independently in order to increase signal to noise ratios and improve the detection and the quantification of peptide fragments. Several approaches currently perform such MS2 refinement for DIA. These include DIA-Umpire (11), Spectronaut (10), SWATHProphet (12), NOFI (13), TargetedMSQC (14), Encyclopedia (15), and Avant-garde (16). The improved quality of quantitative information after the interference correction can be relevant for projects such as those focusing on posttranslational modifications or peptidomics (2).

Traditionally in SWATH-type DIA, quantification relies on the MS2 information. An (optionally recorded) survey scan is followed by consecutive DIA segments covering the entire *m/z* range (9, 17). These settings are inherited from the initial development of SWATH-type DIA on Triple TOF instruments with moderate resolution, combined with the targeted data analysis strategy borrowed from selected reaction monitoring (*e.g.* in Spectronaut (10), OPENSWATH (18), Skyline (18)) and based on mProphet (19). Some researchers have also explored the value of MS1-level signals. Schilling *et al.* introduced MS1 data extraction from DIA with filtering and quantification but did not implement a procedure for characterizing the associated FDR, thereby limiting it to experiments where the numbers of peptides and runs are small enough to facilitate manual inspection (20). Rardin *et al.* (2) performed an exploratory analysis of MS1 and MS2 extracted ion currents of SWATH DIA data and found a strong quantitative correlation between the two. Specifically, they found that MS1 information can be especially relevant for studies of posttranslational modifications. DIA-Umpire (11) extracts the MS1 and MS2 information during a direct analysis of DIA data from a fasta database and correlates this information for identification using a search engine. In the Spectronaut software suite, MS1 and MS2 are fully implemented for identification and quantification (10).

Since recent progress has produced new MS instrumentation with higher resolution at faster speed, both the identification and the quantification of peptides in DIA can benefit from the increased quality of MS1 (21, 22). In particular, high quality and quantitative MS1 and MS2 data now enable statistical inference of differential peptide and protein abundance. While this can be done separately for MS1 or MS2 (as *e.g.* is currently done in Spectronaut), it may be advantageous to simultaneously model MS1 and MS2 quantitative signals by viewing them as technical replicates from the same biological samples (Fig. 2*A*). To the date, the value of a direct join statistical modeling of MS1 and MS2 information for detecting differentially abundant proteins has not been systematically attempted and evaluated. This is due in part to the lack of controlled DIA datasets that make available both MS1 and MS2 quantitative information.

This manuscript contributes a statistical approach for the detection of differential abundant proteins that systematically leverages both MS1 and MS2 information. We applied this procedure to six sets of controlled mixtures. This was including mixtures with realistic biological background variation, and recorded on various instruments. A comparison of the performance of the MS1-MS2-combined method to the individual MS1- or MS2-based methods found consistent improvement in our ability to detect differentially abundant proteins, as judged by the quality of the candidate lists. The influence of the quality of the MS1 data was apparent, enabling the generation of optimal DIA methods for the combined use of MS1 and MS2. Finally, we applied the MS1-MS2-combined method to a clinical investigation and demonstrated that the MS1-MS2-combined method increased the coverage of known activated pathways.

## EXPERIMENTAL PROCEDURES

### 

#### 

##### Overview

We evaluated the impact of the use of MS1 and MS2 quantitative information in DIA using multiple datasets. To ensure the generality of the evaluation, we relied on six diverse sets of controlled mixtures with known ground truth and on a clinical investigation of lung cancer. Specifically, one set of controlled mixtures had defined spike-in of few proteins in a constant background (Spike-in-HEK293-OT dataset). Additionally, a set of controlled mixtures had defined spike-in of few proteins in a background with realistic biological variation (Spike-in-biol-var-OT) (23). The third type consisted of sets of controlled mixtures at defined ratios (MP-LFC-OT, MP-SFC-OT, MP-LFC-TTOF, and MP-LFC-MS1var-OT). Finally, we evaluated the MS1-MS2-combined method in a clinical investigation, comparing healthy tissues to tissues with lung cancer (BiolDS-OT). Data from these experiments were acquired on different instruments of two main classes (time of flight and Orbitrap). The quality of the candidate lists resulting from the statistical testing was used as a criterion for performance evaluation.

##### Sample Preparation for the Controlled Dataset with Biological Background Variation/Spike-in-biol-var-OT

To generate samples for the controlled mixtures with biological background variation (Spike-in-biol-var-OT), 25 mouse cerebellum samples were ordered from AMS Biotechnology (Abingdon, UK). For tissue lysis, half of a cerebellum was lysed in reducing lysis buffer (8 m urea, 0.1 m ammonium bicarbonate, 10 mm TCEP) in a bead mill (3 × 30 beats per second for 30 s, TissueLyser II, Qiagen, Hilden, Germany). To shear the DNA, lysates were sonicated in a Bioruptor (Diagenode, Seraing, Belgium) for five cycles at a high intensity (30 s on, 30 s off). The lysates were cleared by centrifugation (20 min, 16,000 × *g*). 60 μl of the lysate were used for digestion. For the alkylation of the samples, 60 μl of alkylation buffer (8 m urea, 0.1 m ammonium bicarbonate, 40 mm CAA) were added, and the samples were incubated for 1 h at 37 °C. Subsequently, samples were diluted with 600 μl of 0.1 m ammonium bicarbonate buffer including 5 μg of trypsin. Digestion was carried out overnight at 37 °C and constant shaking at 500 rpm. To stop the digestion, samples were acidified with 20% TFA. Peptide mixtures were purified using 96-well plate clean-up plates (NEST group, Southborough, MA) following the manufacturer's protocol. The samples were completely dried by vacuum centrifugation and resuspended in solvent A (1% acetonitrile, 0.1% formic acid in water) including iRT peptides (Biognosys, Schlieren, Switzerland). The UPS2 standard (Sigma, St Louis, MO) was digested separately using the same protocol, but MicroSpin columns were used for the cleanup (NEST group).

The concentrations of the cerebellum samples were adjusted to 1 μg/μl. Next, the samples were spiked five different concentrations of UPS2 standard, each in five different cerebellum samples (in total 25). Based on the lowest abundant UPS2 proteins, the spike-in concentrations were (assuming no losses during UPS2 sample preparation): S1: 0.75 amol/μl, S2: 0.83 amol/μl, S3: 1.07 amol/μl, S4: 2.04 amol/μl, and S5: 7.54 amol/μl.

For library generation, aliquots from all spiked samples were pooled and basified using ammonium hydroxide. The peptide pool was separated by high pH reverse-phase chromatography on a 2.1*150 mm Acquity CSH 1.7-μm column (Waters, MA) using a Dionex Ultimate 3000 LC (Thermo Scientific, Sunnyvale, CA) by a 30-min nonlinear gradient from 1% buffer B (100% acetonitrile)/99% buffer A (20 mm ammonium formate, pH 10) to 40% buffer B. A fraction was taken every 45 s and pooled into 10 final fractions.

##### Preparation of the Samples for the Controlled Dataset with Varying MS1 Resolution/MP-LFC-MS1var-OT

To generate the controlled mixtures with varying MS1 resolution (MP-LFC-MS1var-OT), HeLa, *Caenorhabditis elegans* and *Saccharomyces cerevisiae* digests were prepared as described before (21). Subsequently, two samples were generated with HeLa constant, *C. elegans* 30% change and *S. cerevisiae* 100% change to generate the samples for the controlled dataset with varying MS1 resolution (MP-LFC-OT).

##### Preparation of the Lung Cancer Samples/BiolDS-OT

For the clinical cancer dataset (BiolDS-OT), 12 nonsmall cell lung cancer and 12 matching normal adjacent tissue were purchased from Proteogenex (Culver City, CA). Around 30 mg per tissue were cut and lysed in lysis buffer (8 m urea, 0.1 m ammonium bicarbonate) in a bead mill (3 × 30 beats per second for 30 s, TissueLyser II, Qiagen, Hilden, Germany). DNA was digested by benzonase (Sigma-Aldrich, St. Louis, MO) treatment according to manufacturer's instructions. Lysates were cleared by centrifugation (20 min, 16,000 × *g*). 80 μl of the lysate were used for digestion. For reduction and alkylation of the samples, 80 μl of reduction/alkylation buffer (8 m urea, 0.1 m ammonium bicarbonate, 10 mm TCEP, 40 mm CAA) were added, and the samples were incubated for 1 h at 37 °C. Subsequently, the samples were diluted with 500 μl of 0.1 m ammonium bicarbonate buffer including 10 μg of trypsin. Digestion was carried out overnight at 37 °C and constant shaking at 500 rpm. To stop the digestion, samples were acidified with 20% TFA. Peptide mixtures were purified using 96-well plate cleanup plates (NEST group) following the manufacturer's protocol. The samples were completely dried by vacuum centrifugation and resuspended in solvent A (1% acetonitrile, 0.1% formic acid in water) including iRT peptides (Biognosys). Prior LC-MS analysis peptide concentrations were adjusted to 1 μg/μl. For library generation, aliquots from all samples were pooled (in total 200 μg) and basified using ammonium hydroxide. The peptide pool was fractionated by high pH reverse phase chromatography as described above. Pooled fractions were completely dried by vacuum centrifugation and resuspended in solvent A (1% acetonitrile, 0.1% formic acid in water) including iRT peptides (Biognosys).

##### LC/MS Acquisition of Spike-in-biol-var-OT, MP-LFC-MS1var-OT and BiolDS-OT

For DDA and DIA, 2 μg of each sample were separated using a self-packed analytical PicoFrit column (75 μm × 50 cm length) (New Objective, Woburn, MA) packed with ReproSil-Pur 120A C18-AQ 1.9 μm (Dr. Maisch GmbH, Ammerbuch, Germany) with a 2-h segmented gradient using an EASY-nLC 1200 (Thermo Scientific). The datasets were acquired in a block randomized manner. The Spike-in-biol-var-OT and the MP-LFC-MS1var-OT were acquired on a Q Exactive HF mass spectrometer (Thermo Scientific) with methods modified from (21). The BiolDS-OT dataset was acquired on a Q Exactive HF-X mass spectrometer. The DIA method contained 43 DIA segments of 30,000 resolution with injection time set to auto and automatic gain control of 3*10^6^ and a survey scan of 120,000 resolution with 60ms max injection time and automatic gain control of 3*10^6^. The mass range was set to 350–1650 *m/z*. The default charge state was set to 3. Loop count 1 and normalized collision energy stepped at 25.5, 27, and 30. For the dataset with varying MS1 resolutions, the number of DIA segments was adapted to maintain a constant method cycle time. For the acquisition of the fractionated sample for the library, a DDA method was applied. The TOP15 method was modified from (24) (MS-Methods.xlsx).

##### Mass Spectrometric Data Analysis of Spike-in-biol-var-OT, MP-LFC-MS1var-OT and BiolDS-OT

DIA data were analyzed with Spectronaut Pulsar X 12.0.20491.6, (Biognosys (10)). The default settings were used for the targeted analysis of DIA data in Spectronaut. The initial mass tolerance for MS1 and MS2 was 15 ppm. High precision iRT calibration was used (25). The analysis was performed with and without the built-in interference correction (10). The FDR was calculated according to (19). The DDA spectra were analyzed with the MaxQuant (Version 1.5.6.5) analysis software (26, 27) using default settings (Trypsin/P, two missed cleavages). The search criteria included carbamidomethylation of cysteine as a fixed modification and oxidation of methionine and acetyl (protein N terminus) as variable modifications. The initial mass tolerance for the precursor was 4.5 ppm and for the fragment ions was 20 ppm. The Spike-in-biol-var-OT DDA were searched against the mouse isoform UniProt fasta database (state 11.12.2014, 24,723 entries) and the Biognosys iRT peptides fasta database (uploaded to the public repository). The DDA from the BiolDS-OT dataset was searched against the UniProt fasta database (state 11.12.2014, 20,215 entries) and iRT fasta. The library was generated in Spectronaut by importing the MaxQuant search results using the default settings. Supplemental Table S1 shows the number of entries in the libraries.

##### Analysis of the Spike-in-biol-var-OT Dataset

The dataset was normalized in Spectronaut Pulsar X by the default normalization option. The blood proteins ALBU_MOUSE, ALBU_HUMAN, TRFE_MOUSE, and TRFE_HUMAN, were removed due to the interference between the spiked-in proteins and the background proteins. Precursor and protein FDR were set to 1%. Since the detection limit of MS1 signal was around 100, normalized MS1 intensities below 100 were considered as missing values. Precursors with any missing MS1 intensities or MS2 intensities over all the MS runs were filtered out.

##### Analysis of the Spike-in-HEK293-OT Dataset

The published controlled dataset from Bruderer *et al.* (10) was analyzed with Spectronaut Pulsar X using default settings using the published library. The dataset was normalized in Spectronaut Pulsar X by the default normalization option. Shared peptides of the spike-in proteins with the human background were removed. Four proteins, P07724, Q921I1, P02768, and P02787, were removed due to the interference between the spiked-in proteins and the background of human proteins. The three replicates from group S8 were not used for statistical testing (28). Next, the data were filtered by FDR and intensity thresholds like the Spike-in-biol-var-OT dataset.

##### Analysis of the MP-LFC-OT, MP-SFC-OT, MP-LFC-TTOF, MP-LFC-MS1var-OT, and BiolDS-OT Datasets

The published mixed proteome controlled datasets MP-LFC-OT and MP-SFC-OT from Bruderer *et al.* (21) and MP-LFC-TTOF from Navarro *et al.* (29) were analyzed with Spectronaut Pulsar X using default settings. The MP-LFC-MS1var-OT dataset was analyzed with Spectronaut Pulsar X using default settings using the spectral libraries from Bruderer *et al.* (21). The BiolDS-OT dataset was analyzed with Spectronaut Pulsar X using default settings using the project spectral library described above. All the datasets (MP-LFC-OT, MP-SFC-OT, MP-LFC-TTOF, MP-LFC-MS1var-OT, and BiolDS-OT) were normalized based on housekeeping proteins using a global median approach for MS1 and MS2 separately (30) (supplemental Table S2). Shared peptides between the proteomes were removed. Next, the data were filtered by FDR and intensity thresholds, as in the Spike-in-biol-var-OT dataset.

##### Statistical Modeling

For a given protein, let *X_iprg_* be the *log*_2_ intensity of peptide precursor ion *p* in replicate *r* from group *g*, where *i*ϵ {1, 2}, *p*ϵ {1, …, *P*}, *r*ϵ {1, …, *R*}, and *g*ϵ *{1*, …, *G}.* The index *i* = 1 indicates that *X*_1_*_prg_* is estimated from MS1 signal, and *i* = 2 indicates that *X*_2_*_prg_* is estimated from MS2 signal.

The methods of statistical analysis are summarized in supplemental Fig. S1. When separately analyzing MS1-based quantification, we first summarized the protein abundances in an LC-MS run, by calculating the median MS1 intensities across all its matching peptide ions. In other words, the summarized MS1 intensity of one protein is *Z*_1_*_rg_* = median*_p_*{*X_1prg_*}, the median of all *X*_1_*_prg_* across all peptide ions *p*, in replicate *r* from group *g.* Next, since all the experiments in this manuscript had a multiple group design, we fit a one-way analysis of variance (ANOVA) model:

(1) $$\begin{array}{c} Z_{1rg}=\mu +Group_{1g}+\varepsilon_{1rg}\\ \sum_{g=1}^{G}Group_{1g}=0\\ \varepsilon_{1rg}∼N\left(0,\sigma_{1}^{2}\right) \end{array}$$

In this notation, *Group*_1_*_g_* describes the deviation of the MS1-based expected protein abundance in group *g* from the average expected protein abundance in all the groups. This term is of the main interest in MS1-based quantification. The term ε_1_*_rg_* is the random experimental error with mean 0 and a constant variance. Hypothesis testing for differential abundance was performed based on this model. *P*-values of all the proteins were adjusted for multiple testing by the method of Benjamini and Hochberg (31).

We analyzed MS2-based quantification using the same method (supplemental Fig. S1).

(2) $$\begin{array}{c} Z_{2rg}=\mu +Group_{2g}+\varepsilon_{2rg}\\ \sum_{g=1}^{G}Group_{2g}=0\\ \varepsilon_{2rg}∼N\left(0,\sigma_{2}^{2}\right) \end{array}$$

Here *Z*_2_*_rg_* is the summarized MS2 intensity of the same protein, *i.e. Z*_2_*_rg_* = median*_p_*{*X*_2_*_prg_*}, the median of all *X_2prg_* across all peptide ions *p*, in replicate *r* from group *g. Group*_2_*_g_* describes the deviation of the MS2-based expected protein abundance in group *g* from the average expected protein abundance in all the groups. This term is of the main interest in MS2-based quantification. The term ε_2_*_rg_* is the random experimental error with mean 0 and a constant variance. *p*-values of all the proteins were also adjusted for multiple testing by the method of Benjamini and Hochberg.

In order to jointly analyze the MS1 and MS2 precursor intensities in a statistical model, we first separately normalized MS1 and MS2 intensities of each peptide precursor ion to have zero median across all the replicates. That is, *X′_iprg_* = *X_iprg_* − median*_rg_*{*X_iprg_*} is the normalized *log_2_* intensity of peptide precursor ion *p* in replicate *r* from group *g.* Then normalized protein intensity *Z′_irg_* = median*_p_*{*X′_iprg_*}, the median of all *X'_iprg_* across all peptide ions *p*, in replicate *r* from group *g*. Next, we extended the ANOVA models in Equations (1) and (2) above, to express both MS1 and MS2 signals.

(3) Z′irg+μ+MSi+Groupg+Re⁡plicater(g)+εirg∑i=12MSi=0∑g=1GGroupg=0Re⁡plicater(g)∼N(0,σR2)εirg∼N(0,σ2)

In this notation *MS_i_* is the contribution of MS1 signal and MS2 signal to the estimate of protein abundance *Z′_irg_. Group_g_* describes the deviation of the expected protein abundance in group *g* from the average expected protein abundance in all the groups, on average over MS1 and MS2 signals. This term is of the main interest in joint MS1- and MS2-based quantification. *Replicate_r_*_(_*_g_*_)_ expresses the variability of protein abundance in replicate *r* within group *k*. ε*_irg_* is the random experimental error with mean 0 and a constant variance. As above, hypothesis testing for differential abundance was performed based on this model. Importantly, the combination of MS1 and MS2 signals allows us to distinguish the sources of biological and technological variation (supplemental Fig. S1). *p*-values of all the proteins were adjusted for multiple testing by the method of Benjamini and Hochberg.

We evaluated the performance of the statistical methods in the six controlled mixtures by sorting the candidate lists by *p*-value and examining the true positives as a function of candidate list length. Additionally, for simpler visualization, cuts were taken at fixed candidate list lengths (Top200 for spike-in and Top2000 for proteome mixtures). Finally, the candidate lists were analyzed in terms of the number of true positives and false positives determined based on the ground truth. The BiolDS-OT was evaluated by comparing the candidate lists against an independent study of the same cancers in lung (32). The R script for the analysis was uploaded to the proteomeXchange repository.

## RESULTS

### 

#### 

##### Separately Characterizing the MS1 and MS2 Quantification in DIA

Before integrating the MS1 and MS2 in the statistical modeling, we sought to separately characterize the strengths and weaknesses of the quantitative information of MS1 and MS2. In each dataset, we visually inspected the peptide precursor and fragment signals of the truly differentially abundant proteins. We compared the precision, accuracy, and correlation of precursor quantification by MS1 and MS2 globally across all the proteins.

**Fig. 2.**
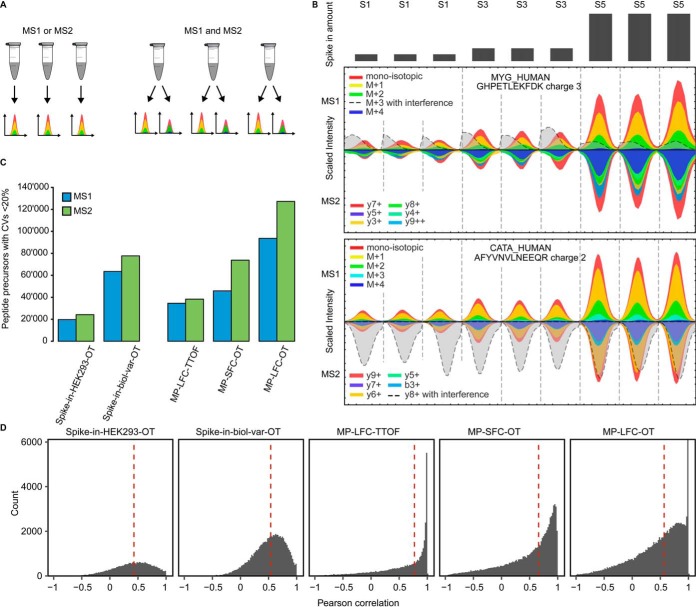
**MS1 and MS2 quantification characteristics in DIA** (*A*) The MS1 and MS2 quantitative signals can be viewed as technical replicates from the same biological samples. (*B*) Extracted ion currents of two peptides derived from spike-in proteins from the Spike-in-biol-var-OT dataset of sample 1, 3, and 5. The interferences were manually identified as not following the predefined pattern of differential abundance. (*C*) The CVs for all precursors on condition level were calculated for the controlled datasets separately for MS1 and MS2 and separately for each condition. The graph displays the counts of precursors with CVs below 20%. (*D*) Pearson correlation between the precursor abundances in MS1 and MS2 space in the controlled datasets. The median is indicated by the red dotted line.

Visual inspection of the DIA data using Spectronaut showed that the MS1 and MS2 interferences occur independently. In the controlled mixtures, interferences of peptide precursors with differential abundance can be clearly spotted because they do not follow the expected patterns of abundance between conditions. In an example with interference on MS1, a coeluting peptide was of the same *m/z* (Fig. 2*B*, *upper panel*) In an example with interference on MS2, a fragment of a coeluting and cofragmented peptide precursor was of the same mass (Fig. 2*B*, *lower panel*).

To globally assess the precision of MS1 and MS2 quantification, we calculated the CVs on the precursor level for the controlled mixtures within conditions. For all the tested datasets, the precision of MS2 quantification was higher than that of MS1, when counting precursors with CVs <20%. The number of precursors with CVs below 20% (on MS2) was between 11 and 61% higher than MS1 (Fig. 2*B*). Consistently, the medians of the CVs of MS2 quantification were consistently lower than those of MS1 (supplemental Fig. S3*A*). The CVs of precursors on MS1 had a median between 9% and 25%, and CVs on MS2 it was between 7% and 15%.

In the controlled mixtures based on mixed proteomes acquired on the Orbitrap instruments, the accuracy of fold change estimation was similar between MS1 and MS2 (supplemental Fig. S3*B*). In the MP-LFC-TTOF dataset recorded on the time of flight instrument, the fold change was more compressed in the MS1 space than in MS2, while the fold change estimate of the combined MS1 and MS2 information was between the estimates of the individual (supplemental Fig. S3*B*). The Pearson correlation between MS1 and M2 quantification had a median value of 0.5 (Fig. 2*C*). These analyses demonstrated that, although MS1 and MS2 are of generally similar quality, they can be of variable quality for specific analytes.

##### Combining the Quantitative Information in MS1 and MS2

**Fig. 3.**
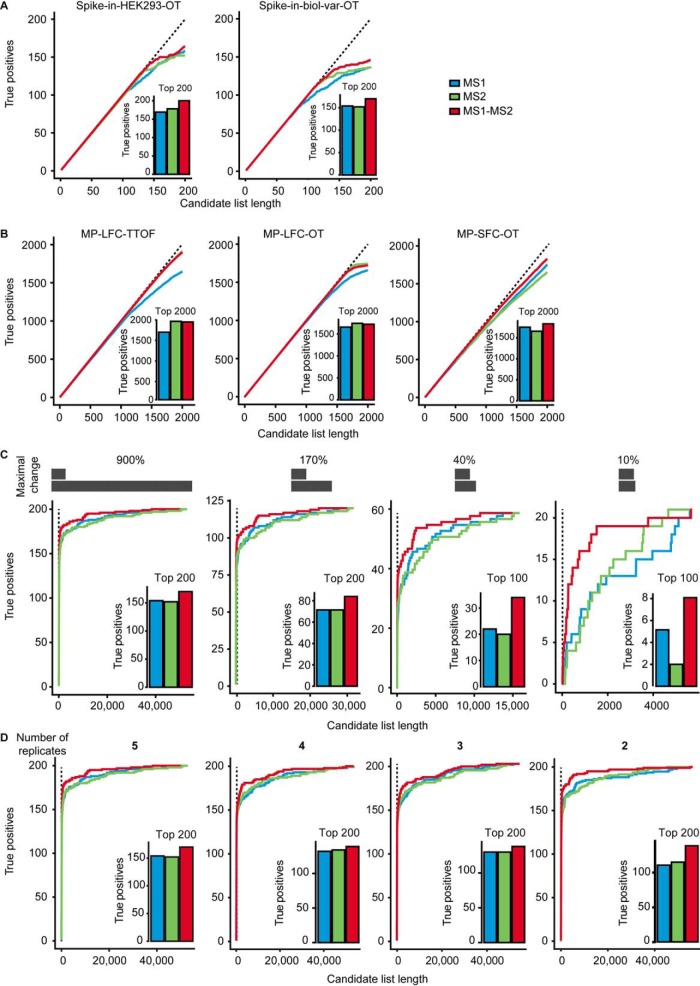
**Benchmarking of the MS1-MS2 combined method** (*A*) Statistical inference of differential abundance was performed for spike-in datasets. The 200 proteins with the smallest adjusted *p* values were sorted by their *p* value. Next, the number of true positive differentially abundant proteins was displayed as a function of the candidate list containing true and false positives. The dotted line indicates a perfect candidate list containing only true positives (slope = 1). *Inset*: the number of true positives in the list of 200 proteins with the smallest adjusted *p* values. (*B*) As in (*A*), but for the mixed proteome datasets as in (*A*). (*C*) Statistical detection of differentially abundant proteins was performed as above for subsets of the Spike-in-biol-var-OT dataset with decreasing maximal true fold change, by selecting subsets of the dataset. The first plot to the left is based on the samples 1 to 5, the second to the left on 1 to 4, the third from the left on 1 to 3, and the right plot shows 1 to 2. The resulting candidate lists were analyzed as above. (*D*) Statistical detection of differentially abundant proteins was performed as above for subsets of the Spike-in-biol-var-OT dataset with decreasing numbers of replicates. The resulting candidate lists were analyzed as above.

Our next step was to evaluate the ability of the proposed MS1-MS2-combined method to statistically detect differentially abundant proteins in the controlled mixtures. First, we compared the true positive and false positive differentially abundant proteins (as defined by the ground truth), detected by MS1 alone, by MS2 alone, and by the MS1-MS2-combined method. The MS1-MS2-combined method always outperformed the individual tests (Fig. 3*A*, supplemental Fig. S4*A*, supplemental Table S4, and statistical-inference-results.zip). In the controlled mixtures with spike-in proteins, the top differentially abundant proteins consisted mostly of the true positives (the number of true positives among the top 200 differentially abundant proteins were as follows. Spike-in-HEK293-OT, MS1: 129, MS2: 160, and MS1-MS2: 164; Spike-in-biol-var-OT, MS1: 72, MS2: 111, and MS1-MS2: 113). In the proteome mixtures, the performance of the MS1-MS2-combined method was similar or better than the individual (Fig. 3*B*, supplemental Fig. S4*B*, supplemental Table S4, and statistical-inference-results.zip).

In order to distinguish the role of increasing the number of replicates from that of reducing the undesirable effects of interferences in MS1 or MS2 signals, we reanalyzed the datasets with and without interference correction implemented in Spectronaut. The interference correction improved the statistical power in all the datasets (supplemental Fig. S5*A*). The combination of MS1 and MS2 information could mitigate the negative impact of interferences. The performance of the combined method without interference correction was comparable to the performance of MS2 alone with interference correction in all but one set of controlled mixtures (supplemental Fig. S5*B*).

Next, we analyzed the effect of the magnitude of the fold changes on the performance of the statistical models. We split the pairwise comparisons of the Spike-in-biol-var-OT into subsets with decreasing maximal fold changes from 900% to 10% (Fig. 3*C*). Additionally, for the controlled mixtures Spike-in-HEK293-OT and MP-SFC-OT (supplemental Fig. S6), the MS1-MS2-combined method performed better with smaller fold changes than the individual MS1- or MS2-based quantification. Detecting small fold changes proved challenging because the observed maximum of true positives was reached more slowly in all the three methods. This challenge also manifested itself by the earlier deviation from the perfect candidate list.

We further evaluated the effect of replication on the outcome of the statistical analyses. We used the three statistical approaches to analyze the Spike-in-biol-var-OT dataset with a decreasing number of replicates. The MS1-MS2-combined method maintained higher statistical power than the individual tests (Fig. 3*D*). At the lowest number of replicates (two), the MS1-MS2-combined method had a 13% better sensitivity as compared with MS1 and 17% as compared with MS2.

An important parameter of DIA is the time allocated to MS1 and MS2, respectively (thus affecting the resolution for Orbitrap instruments). Because current DIA methods have been mostly optimized for MS2-based quantification, we evaluated the influence of the experimental MS1 resolution on an Orbitrap mass spectrometer on the MS1-MS2-combined statistical procedure. The controlled mixture MP-LFC-MS1var-OT was profiled with DIA while varying MS1 resolutions (30,000 to 240,000) and balancing MS2 time (at a constant resolution of 30,000) to keep the cycle time of the methods constant. We then characterized the quantitative precision, as well as the detection of differentially abundant proteins in these settings (supplementary File MP-LFC-MS1var-OT.zip). The DIA method with 120,000 MS1 resolution consistently produced most precursors with CVs below 20%, both for MS1 and MS2 (supplemental Fig. S7*A*). This can be explained by the fact that peak picking and integration in Spectronaut is dependent on MS1 and MS2. For all three statistical models, the best candidate lists were obtained at the 120,000 MS1 resolution. The MS1-MS2-combined approach showed the best overall performance, likely due to a sufficient MS1 resolving power and a balance in the number of MS2 segments (supplemental Figs. S7*B* and S7*C*). This is practical because it corresponds to the widely used DIA methods.

##### Testing Proteins for Differential Abundance in the Set of Clinical Samples with Lung Cancer

**Fig. 4.**
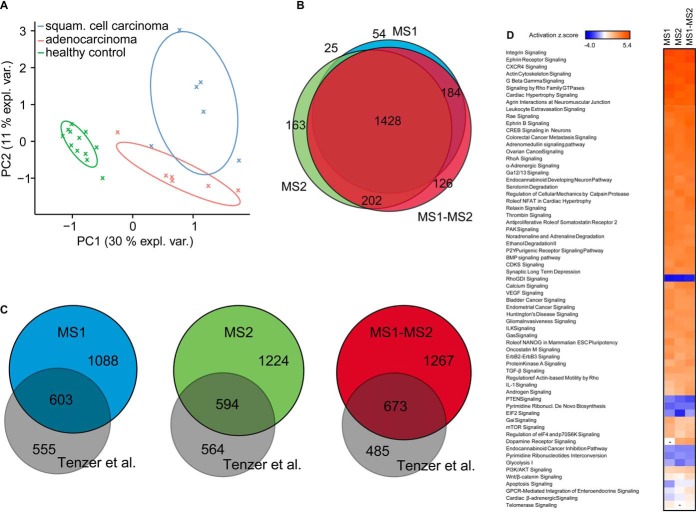
**MS1-MS2-combined method based differential abundance testing in clinical samples** (*A*) 12 healthy lung and 12 cancer (six adenocarcinomas and six squamous cell carcinomas) were analyzed by mass spectrometry. The resulting data were subjected to principal component analysis. (*B*) Statistical detection of differentially abundant proteins was performed with MS1-, MS2-based and the MS1-MS2-combined method. The overlap of differentially abundant proteins (FDR <0.05) was calculated on the protein level. (*C*) The candidate lists from the testing approaches were compared with the candidate list of an independent lung cancer study by Tenzer *et al.* (32). (*D*) The functional analyses were generated through the use of IPA (33). The figure plots the activation states of the pathways according to IPA.

Finally, we evaluated the performance of the MS1-MS2-combined method in a clinical investigation of 12 healthy lung and 12 cancer samples (six adenocarcinomas and six squamous cell carcinomas). We performed an exploratory analysis, statistical analysis of differential abundance using the MS1- or the MS2-based method or the MS1-MS2-combined method (supplementary File BiolDS-OT.xlsx), and biological pathway analysis using ingenuity pathway analysis (IPA) (33). Principal component analysis-based clustering revealed a clear separation of healthy and tumor samples, indicating the biological separation between the two sample sets (Fig. 4*A*). The MS1-MS2-combined method produced the largest list of differentially abundant proteins (multiple testing correction BH, FDR <1%). The three methods shared 65% of the union of differentially abundant proteins (Fig. 4*B*). The MS2-based and the MS1-MS2-combined method showed a large portion of unique candidates, respectively (7.5% and 5.8% of the union). The results of the MS1-MS2-combined method had a larger overlap with the MS1 approach, missing 79 candidates. The MS2-based method missed 238 differentially abundant proteins that were reported by MS1.

Because the true differentially abundant proteins are unknown, we compared our results to the results of an independent study based on the same cancer type by Tenzer *et al.* (32). The results of the MS1-MS2-combined method had the largest overlap with Tenzer *et al.* The overlap had 70 proteins more than the overlap based on the MS2 method (and 79 more than the MS1-based) (Fig. 4*C*). Upon investigation of pathway enrichments in the candidate lists, we found that among the 64 most enriched pathways there was a high degree of overlap between all three different methods. The pathways were consistently either activated or deactivated (Fig. 4*D*). The proteins uniquely identified by the MS1-MS2-combined method belonged to the same pathways, such as the pathways for the shared proteins, indicating a more comprehensive description (supplemental Fig. S8).

## DISCUSSION

In complex mixtures, peptides of the same mass can coelute, and the coeluting peptides can share fragments of the same mass. Modern DIA experiments allow us to characterize these two independent quantitative spaces. The combined use of MS1- and MS2-level information increases the number of technical replicates and, therefore, the precision of the measurement. The improved precision, combined with the ability to separate the sources of biological and technological variation by the statistical modeling, in turn improve the power of detecting differentially abundant proteins.

Moreover, because interferences in MS1 and MS2 usually do not correlate, another strength of the combined use of MS1 and MS2 is in reducing the negative impact of the interferences in one of the quantitative spaces on the downstream statistical analysis (Supplemental Fig. S5).

To take advantage of both layers of quantification, we developed a statistical model that combines the use of MS1 and MS2. We demonstrated the advantages of this approach on multiple sets of controlled mixtures from multiple instrument platforms. We noticed the largest improvement in the detection of differential abundance for small fold changes, a finding that is particularly relevant for plasma studies and in cases of a low number of replicates (34–36). On a clinical investigation of lung cancer, the proteins uniquely identified by the MS1-MS2-combined method increased the coverage of the pathway enriched by MS1- and MS2-based quantification. Despite this improvement, the MS1-MS2-combined method is not a substitute for adequate biological replication.

We believe that the proposed approach may have a high potential in the future as the technology evolves. For example, the recent development of BoxCar MS1 acquisition (37) improves the intrascan dynamic range, which leads to better MS1 quantification. Therefore, it is conceivable that the approach presented here will become even more powerful and will lead to sensitive and robust statistics when combining BoxCar MS1 and DIA.

## Data availability

The raw mass spectrometric data, the spectral libraries, and the quantitative data tables have been deposited to the ProteomeXchange Consortium via the PRIDE partner repository (38) with the dataset identifier PXD016647. The saved projects from Spectronaut can be reviewed with the Spectronaut Viewer (www.biognosys.com/spectronaut-viewer).

## Supplementary Material

MS Methods summary

Supplemental Information

BiolDS-OTstatistical results

Statistical results controlled mixtures

## Footnotes

^1^ The abbreviations used are:

LC-MS: liquid chromatography-mass spectrometry
DDA: data-dependent acquisition
DIA: data-independent acquisition
SWATH: sequential window acquisition of all theoretical fragment ions
MP: mixed proteomes
SFC and LFC: small/large fold change
OT: orbitrap
TTOF: triple quadrupole time of flight
CV: coefficient of variation
BH: Benjamini-Hochberg
FDR: false discovery rate.
