# Supplementary material for: Combining Precursor and Fragment Information for Improved Detection of Differential Abundance in Data Independent Acquisition
Source: Mol Cell Proteomics. 2019 Dec 30;19(2):421–30. doi: 10.1074/mcp.RA119.001705 (PMC7000113; doi:10.1074/mcp.RA119.001705)
Supplement: Supplemental Information [file 154840_1_supp_443469_q2zq36.pdf]

Supplemental Material:

Combining Precursor and Fragment Information for Improved Detection of Differential Abundance in Data Independent Acquisition

Supplementary tables:

Supplemental Table S1: **Spectral library properties**

Data set	Precursors	Peptides	Protein groups
**Spike-in-biol-var-OT**	**170,520**	**120,517**	**8360**
**BiolDS-OT**	**120,693**	**91,264**	**7317**

Supplemental Table S2: **Human housekeeping proteins used for normalization**

**Protein Accession**	
E9PAV3	P62913
O15372	P62937
P04350	P62979
P04406	P63173
P0CG48	P63261
P18124	P68133
P18206	Q14103
P27797	Q15233
P60709	Q53S24
P60866	Q71U36
P60953	Q9GZZ7
P61077	Q9UNX3
P62280	

Supplemental Table S3: **Overview of the experimental datasets in this manuscript**

Dataset	Preprocessing steps	# of measurements	# of precursors	# of proteins	# of differential abundant proteins	Mean # of precursors per protein	missing values precursor	missing values protein
Spike-in-HEK293-OT	Filter precursors based on q-value and MS1 intensity	599,210	29,017	3,161	12	9.18	1.67%	0.2%
	Remove precursors with missing values	558,243	26,583	3,102	11	8.57	0%	0%
Spike-in-biol-var-OT	Filter precursors based on q-value and MS1 intensity	2,416,926	131,699	6,501	32	20.26	26.59%	3%
	Remove precursors with missing values	1,617,775	64,711	5,295	20	12.22	0%	0%
MP-LFC-TTOF	Filter precursors based on q-value and MS1 intensity	251,479	48,699	5,474	3,211	8.90	13.93%	6%
	Remove precursors with missing values	197,760	32,960	4,425	2,178	7.45	0%	0%
MP-LFC-OT	Filter precursors based on q-value and MS1 intensity	829,198	175,533	10,752	4,420	16.33	21.27%	11.2 %
	Remove precursors with missing values	621,876	103,646	8,037	1,861	12.90	0%	0%
MP-SFC-OT	Filter precursors based on q-value and MS1 intensity	706,398	165,068	11,867	6,829	13.91	28.68%	4.8%
	Remove precursors with missing values	449,952	74,992	9,496	5,390	7.90	0%	0%
BiolDS-OT	Filter precursors based on q-value and MS1 intensity	2,074,693	116,287	7,169	Not available	16.22	25.66%	8.3%
	Remove precursors with missing values	1,026,720	42,780	4,392	Not available	9.74	0%	0%

Supplemental Table S4: **Results of model fitting and testing of proteins for differential abundance** (adjusted p-value <0.01). *PPV* denotes positive predictive value, i.e. the proportion of true positives among the reported differentially abundant proteins. Area under the curve (AUC) is from receiver operating characteristic curves that express the true positive changes in differential abundance as function of the false positives.

Dataset	Quantitative information	AUC	Partial AUC	# of TPs	# of FPs	Sensitivity	Specificity	PPV
Spike-in-HEK293-OT	MS1	0.9118	0.0784	167	130	0.7229	0.9980	0.5623
	MS2	0.9515	0.0854	179	171	0.7749	0.9974	0.5114
	MS1and MS2	0.9769	0.0923	193	128	0.8355	0.9980	0.6012
Spike-in-biol-var-OT	MS1	0.9563	0.0850	142	91	0.7100	0.9983	0.6094
	MS2	0.9475	0.0848	144	151	0.7200	0.9971	0.4881
	MS1and MS2	0.9758	0.0904	158	132	0.7900	0.9975	0.5448
MP-LFC-TTOF	MS1	0.8662	0.0538	1447	205	0.6644	0.9088	0.8759
	MS2	0.9687	0.0835	2033	219	0.9334	0.9025	0.9028
	MS1and MS2	0.9578	0.0822	1952	213	0.8962	0.9052	0.9016
MP-LFC-OT	MS1	0.9528	0.0848	1599	201	0.8592	0.9675	0.8883
	MS2	0.9651	0.0929	1731	80	0.9301	0.987	0.9558
	MS1and MS2	0.9571	0.0916	1710	193	0.9189	0.9688	0.8986
MP-SFC-OT	MS1	0.7385	0.0272	1	0	0.0002	1	1
	MS2	0.6739	0.0236	0	0	NA	NA	NA
	MS1and MS2	0.8082	0.0357	7	0	0.0013	1	1

Supplementary Figures:

	MS1 signal	MS2 signal	Combined MS1 and MS2 signal
Normalization	No	No	Normalize peptide precursor ion intensity X_{iprg} to have zero median across all the replicates<br>$X'_{iprg} = X_{iprg} - median_{rg}(X_{iprg})$
Protein quantification	Median summarization<br>$Z_{1rg} = median_p(X_{1prg})$	Median summarization<br>$Z_{2rg} = median_p(X_{2prg})$	Median summarization for MS1 and MS2 signal individually<br>$Z'_{irg} = median_p(X'_{iprg})$
Statistical modeling	$Z_{1rg} = \mu + Group_{1g} + \varepsilon_{1rg}$<br>$\sum_{g=1}^{G} Group_{1g} = 0$<br>$\varepsilon_{1rg} \sim N(0, \sigma_1^2)$	$Z_{2rg} = \mu + Group_{2g} + \varepsilon_{2rg}$<br>$\sum_{g=1}^{G} Group_{2g} = 0$<br>$\varepsilon_{2rg} \sim N(0, \sigma_2^2)$	$Z'_{irg} = \mu + MS_i + Group_g$ $+ Replicate_{r(g)} + \varepsilon_{irg}$<br>$\sum_{i=1}^{2} MS_i = 0$<br>$\sum_{g=1}^{G} Group_g = 0$<br>$Replicate_{r(g)} \sim N(0, \sigma_R^2)$<br>$\varepsilon_{irg} \sim N(0, \sigma^2)$
Model-based testing between Group *g* and *g'*	$\frac{\bar{Z}_{1.g} - \bar{Z}_{1.g'}}{SE\{\bar{Z}_{1.g} - \bar{Z}_{1.g'}\}} = \frac{\bar{Z}_{1.g} - \bar{Z}_{1.g'}}{\sqrt{\frac{2\sum_{g=1}^{G}\sum_{r=1}^{R}(Z_{1rg} - \bar{Z}_{1.g})^2}{R}}}$<br>$\sim t_{G(R-1)}$	$\frac{\bar{Z}_{2.g} - \bar{Z}_{2.g'}}{SE\{\bar{Z}_{2.g} - \bar{Z}_{2.g'}\}} = \frac{\bar{Z}_{1.g} - \bar{Z}_{1.g'}}{\sqrt{\frac{2\sum_{g=1}^{G}\sum_{r=1}^{R}(Z_{2rg} - \bar{Z}_{2.g})^2}{R}}}$<br>$\sim t_{G(R-1)}$	$\frac{\bar{Z}'_{..g} - \bar{Z}'_{..g'}}{SE\{\bar{Z}'_{..g} - \bar{Z}'_{..g'}\}} = \frac{\bar{Z}'_{..g} - \bar{Z}'_{..g'}}{\sqrt{\frac{2\sum_{g=1}^{G}\sum_{r=1}^{R}(\bar{Z}'_{.rg} - \bar{Z}'_{..g})^2}{R}}}$<br>$\sim t_{G(R-1)}$

Supplemental Fig. S1: **Statistical approaches for detecting differentially abundant proteins.** X_{iprg} is the log_2 intensity of peptide precursor ion *p* in replicate *r* from group *g*. X_{1prg} is estimated from the MS1 signal and X_{2prg} is estimated from the MS2 signal. Z_{irg} is the protein intensity in replicate *r* from group *g*. X'_{iprg} is the normalized log_2 intensity of peptide precursor ion *p* in replicate *r* from group *g*. Z'_{irg} is the normalized protein intensity in replicate *r* from group *g*. $\sum_{g=1}^{G} Group_{1g} = \sum_{g=1}^{G} Group_{2g} = \sum_{i=1}^{2} MS_i = 0$ are the identifiability constraints required for the estimation of the model parameters. σ_R^2 is the between-replicates variance. σ_1^2, σ_2^2 and σ^2 are the variances of the measurement error. All random deviations are independent.

A

Name	Category	Fold change	Conditions	Replicates	Original name	Comment	Published
Spike-in-HEK293-OT	spike-in	Known	8	3	GSDS		Bruderer et al., 2015 MCP
Spike-in-biol-var-OT	spike-in	Known	5	5	n.a.	25 cerebelli background	Prepared for this study
MP-LFC-TTOF	Proteome mixtures	Known	2	3	LFQ		Navarro et al. 2016 Nat. Biotech.
MP-LFC-OT	Proteome mixtures	Known	2	3	LFC		Bruderer et al, 2017 MCP
MP-SFC-OT	Proteome mixtures	Known	2	3	SFC		Bruderer et al., 2017 MCP
MP-LFC-MS1var-OT	Proteome mixtures	Known	2	3	n.a.	aquired with varying MS1 resolutions	Prepared for this study
BiolDS-OT	healthy and cancer samples	n.a.	2	12	n.a.	cancer samples are from two subtypes	Prepared for this study
Abbreviations (OT=Orbitrap, TTOF=Triple-TOF,PM=Proteome mixtures, LFC=large fold change, SFC=small fold change, MS1var=Varying MS1 resolutions)							

B

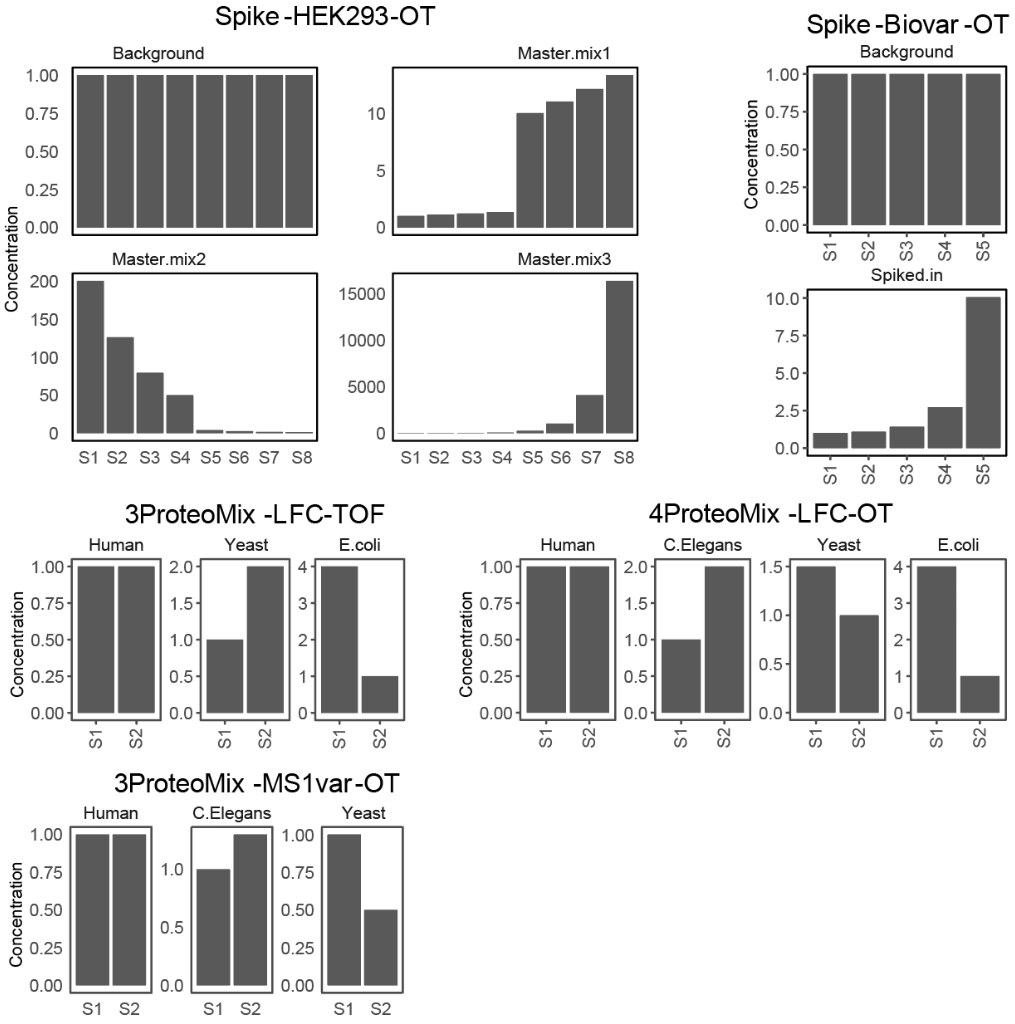

Supplemental Fig. S2 **Overview of the experimental datasets** (A) Overview over the datasets used in this study. (B) Schema of differential abundance in the controlled mixtures.

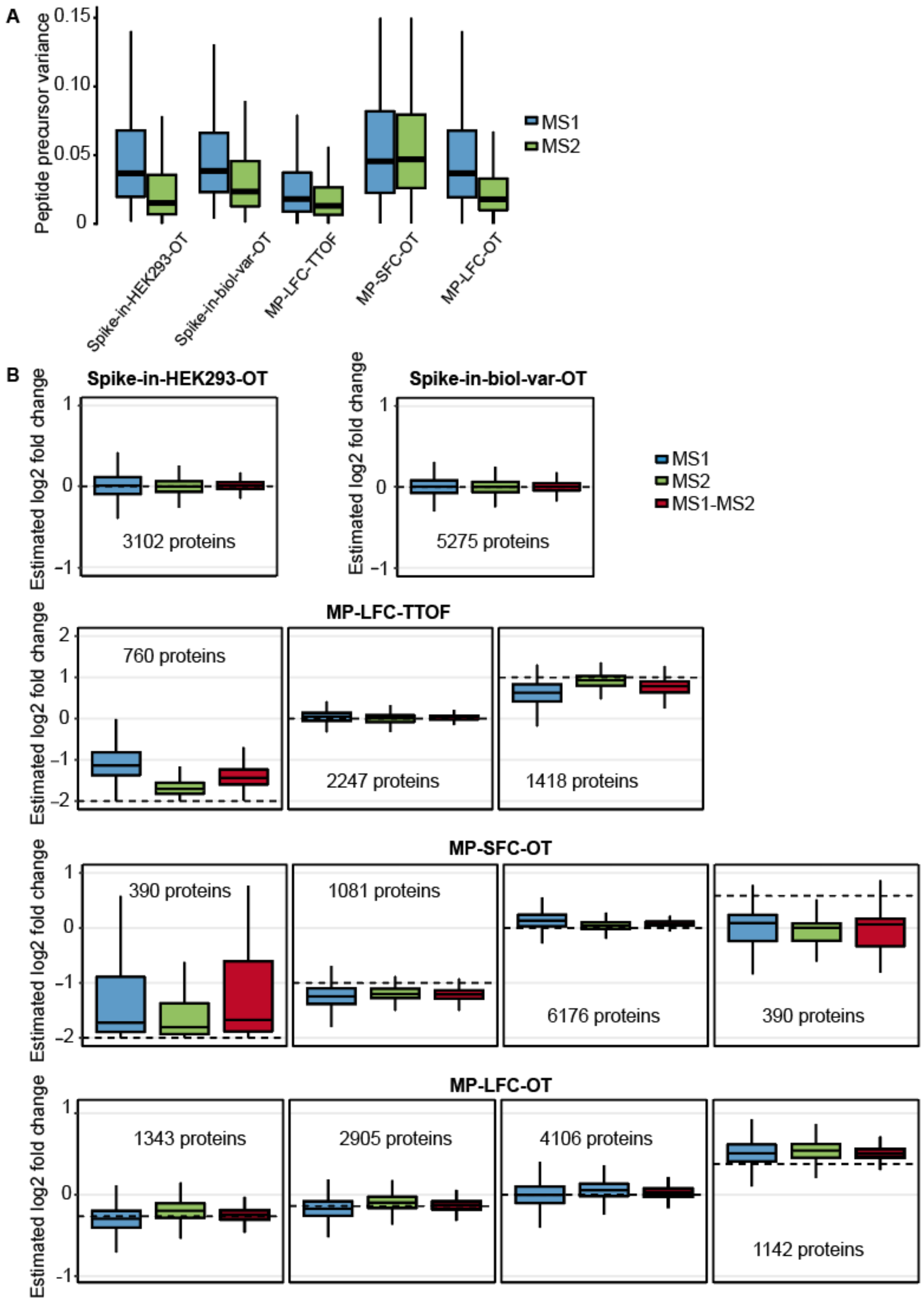

Supplemental Fig. S3: **Coefficients of variation in MS1 and MS2 space.** (A) The variances of the precursors were calculated for the controlled datasets with mixed proteomes. The input for the calculation were the normalized precursor quantities on condition level of the constant background. (B) Estimated log2 fold change for MS1 and MS2 and combined MS1-MS2 in the controlled mixtures. Dashed lines indicate the true values.

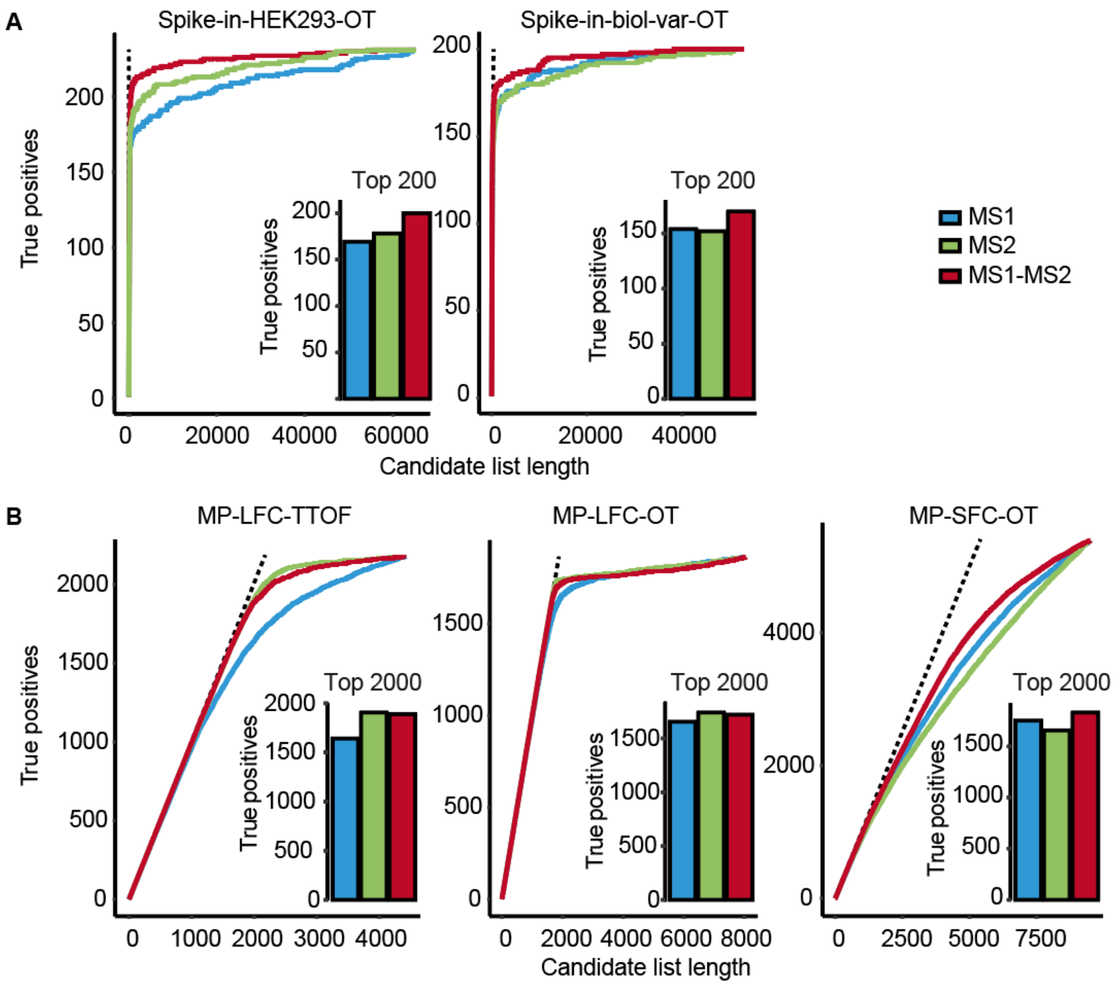

Supplemental Fig. S4: **Benchmarking of the MS1-MS2 combined method on the controlled mixtures.** The controlled mixtures were analyzed using the statistical testing methods. Then, the proteins were sorted by their adjusted p-value. Next, the number of true positive differentially abundant proteins was displayed as a function of number of true and false positives. The dotted line indicates a perfect candidate list containing only true positives (slope = 1). Inset: the number of true positives in the list of 200 proteins with the smallest adjusted p-values. (A) The spike-in datasets. (B) The mixed proteome datasets.

A

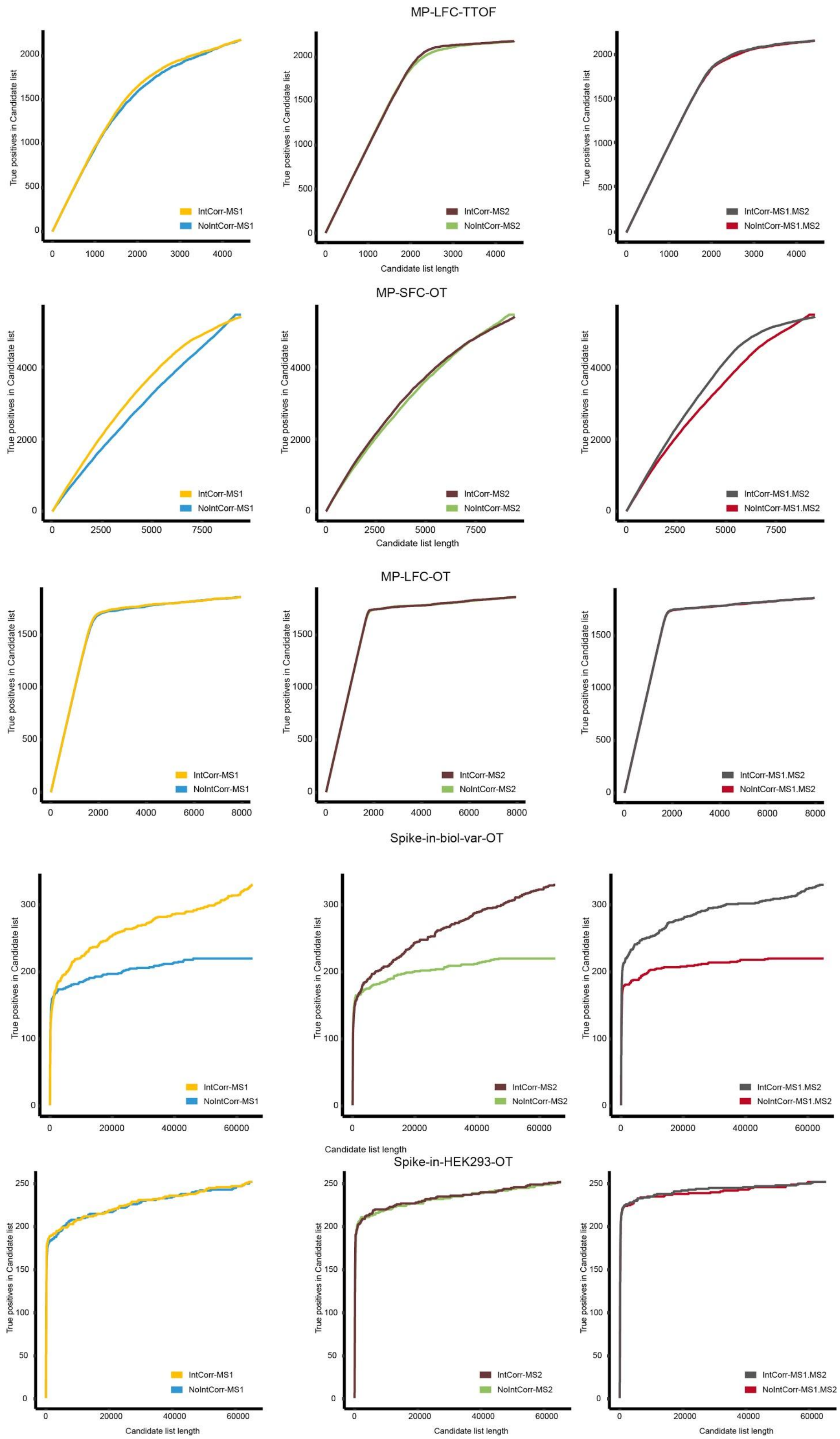
MP-LFC-TTOF
MP-SFC-OT
MP-LFC-OT
Spike-in-biol-var-OT
Spike-in-HEK293-OT
True positives in Candidate list
Candidate list length
IntCorr-MS1
NoIntCorr-MS1
IntCorr-MS2
NoIntCorr-MS2
IntCorr-MS1.MS2
NoIntCorr-MS1.MS2

B

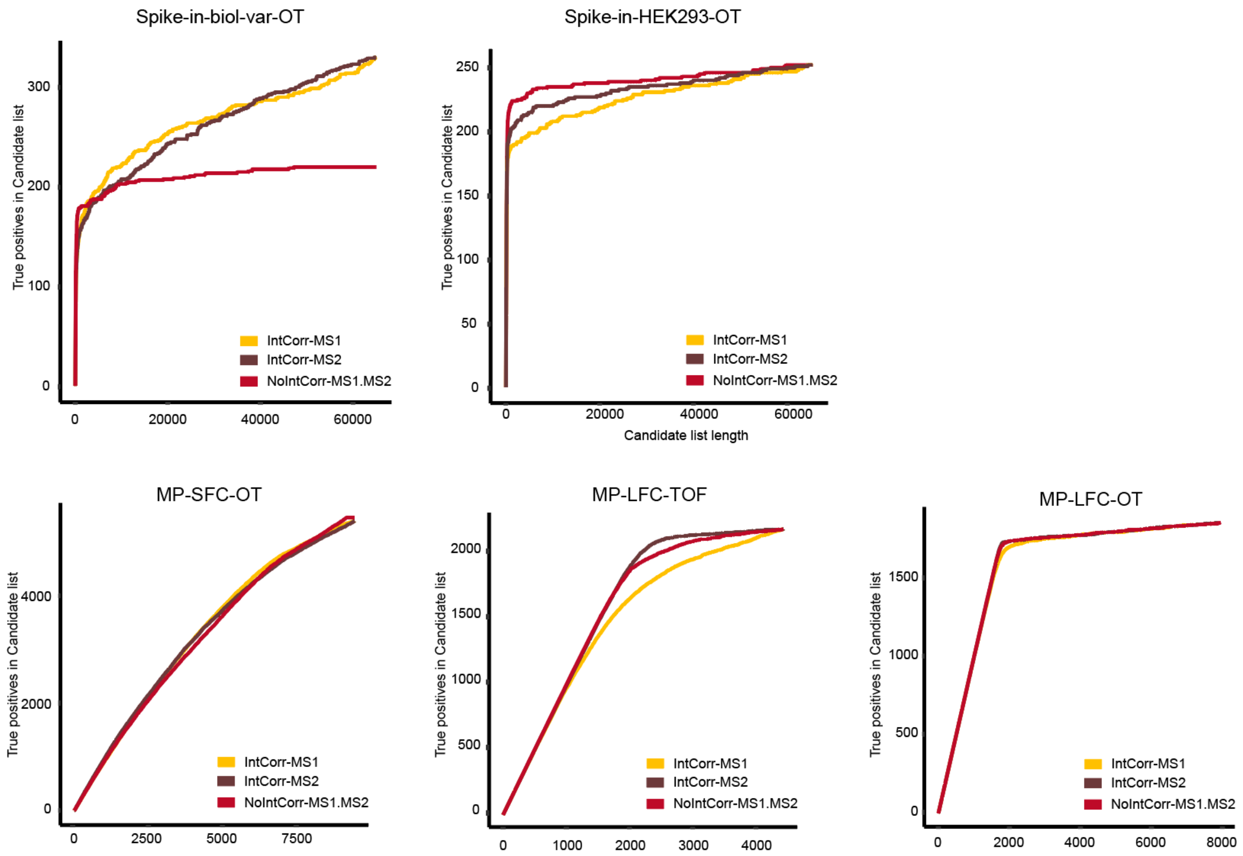

Supplemental Fig. S5: **Statistical analysis of the controlled DIA data sets with and without interference correction of Spectronaut.** (A) The targeted analysis of the controlled DIA datasets was performed in Spectronaut with and without interference correction. Statistical inference of differential abundance was performed for all resulting data tables. The proteins in the candidate lists were sorted by their adjusted p-value. Next, the number of true positive differentially abundant proteins was displayed as a function of subsets of proteins of varying size with the smallest p-values. (B) Comparison of MS1 or MS2 individual methods with interference correction to the MS1-MS2 combined method without interference correction.

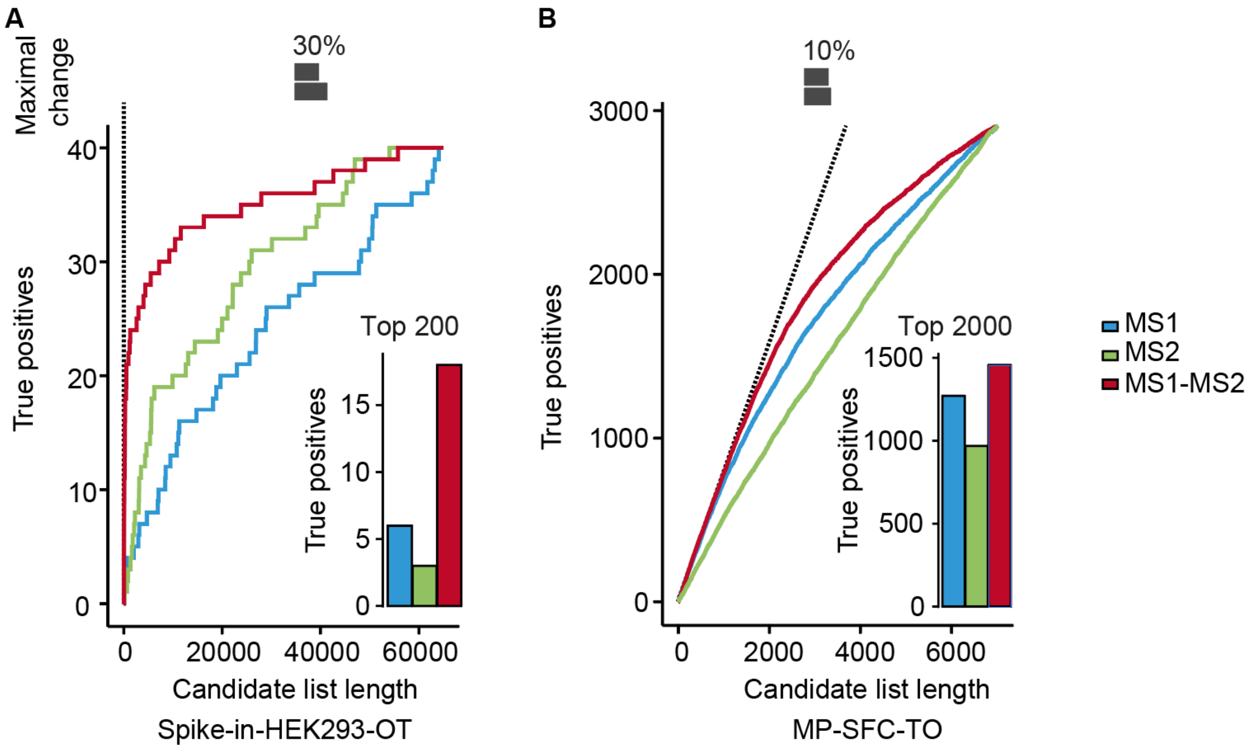

Supplemental Fig. S6: **Statistical inference of differential abundance among a subset of proteins with small fold changes** (A) Statistical inference of differential abundance was performed on a subset of the Spike-in-HEK293-OT with max 30% change in pairwise comparisons. Candidates were sorted by p-value based on the ground truth, the number of true positives in the candidate list was plotted as a function of candidate list length (true positives and false positives). The dotted line indicates the perfect candidate list with only true positives. (B) Statistical inference of differential abundance was performed on a subset of the MP-SFC-OT with a maximum of 10% change in the pairwise comparisons. The dotted line indicates the perfect candidate list with only true positives.

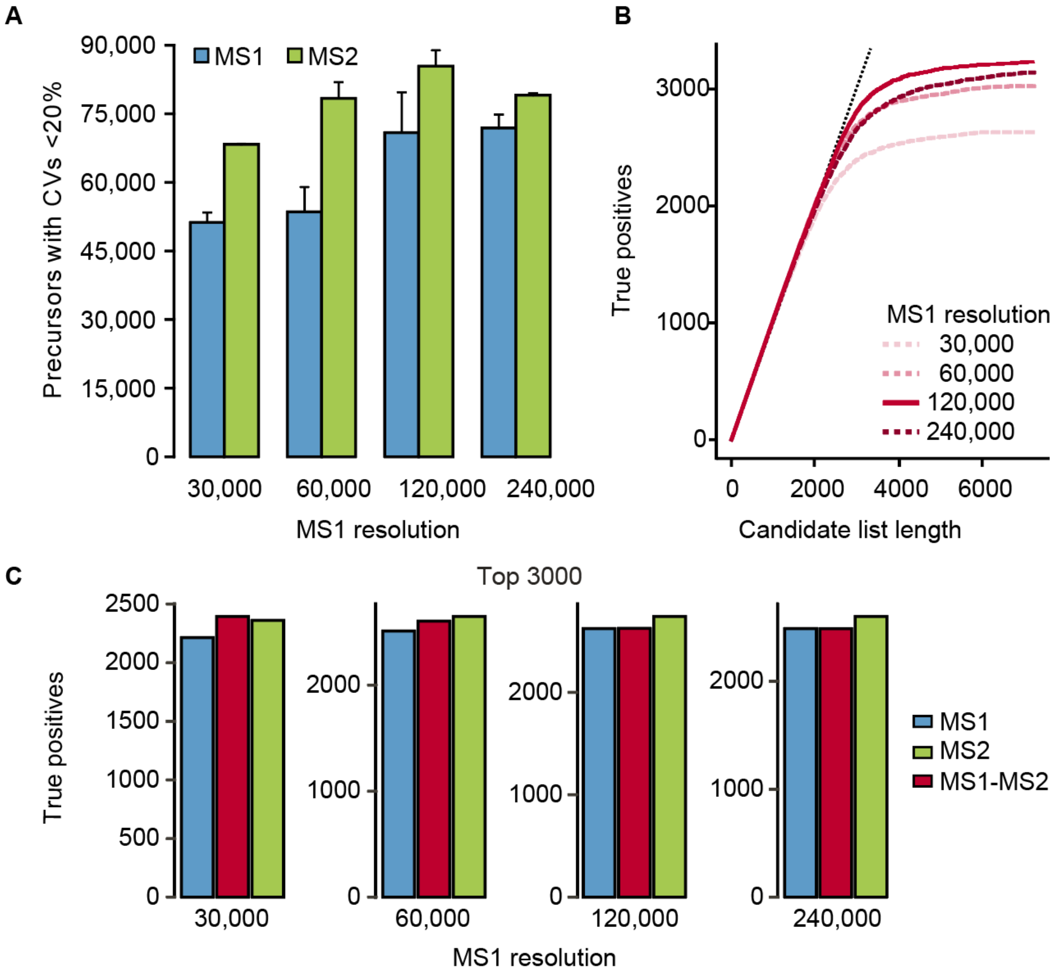

Supplemental Fig. S7: **Impact of varying MS1 resolution in the MP-LFC-MS1var-OT dataset** (A) Number of peptide precursors with CVs<20% for the different MS1 resolution acquisitions of the MP-LFC-MS1var-OT dataset. (B) Detection of differentially abundant proteins with the MS1-MS2-combined method. The proteins in the dataset were sorted by their adjusted p-value. Next, the number of true positive differentially abundant proteins was displayed as a function of subsets of proteins of varying size with the smallest p-values. The dotted line indicates a perfect candidate list containing only true positives. (C) Details of detection of differential abundance for the top 3000 proteins.

A

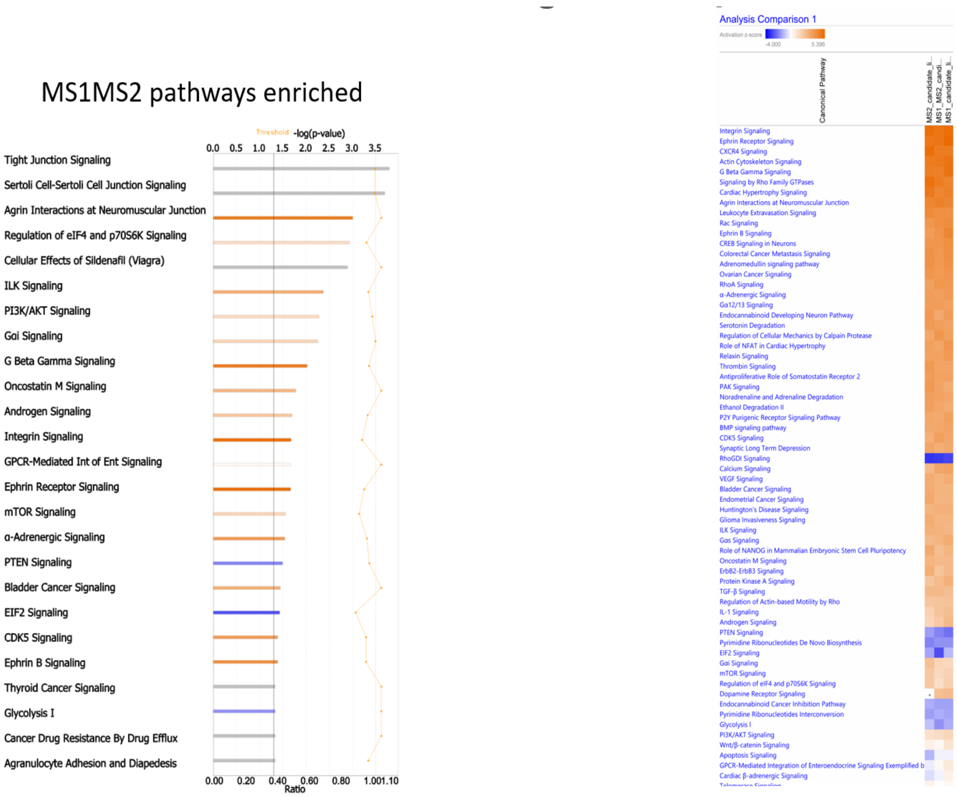

B

MS1MS2 unique proteins pathways enriched

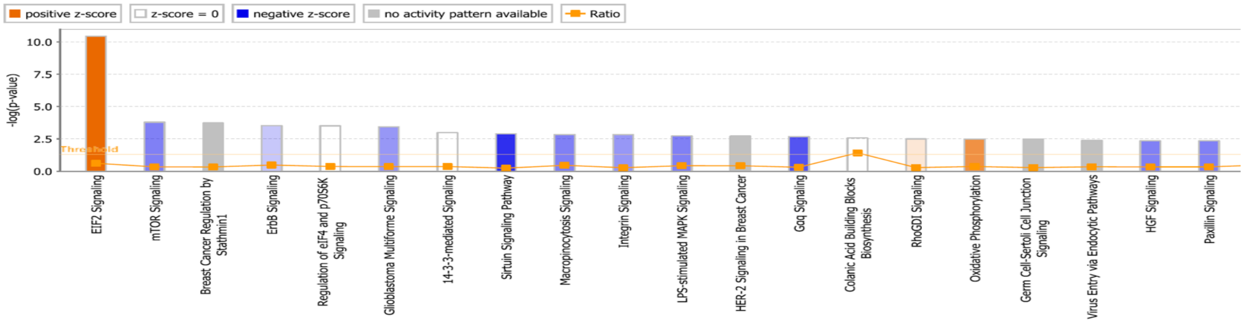

Supplemental Fig. S8: **Pathway enrichment using the MS1-MS2-combined method** (A) Pathway enrichment of all differentially abundant proteins by using IPA and (B) the proteins identified uniquely detected by the MS1-MS2-combined method and not by the individual (MS1 or MS2). The activation state of the pathways is indicated by the blue and orange colors according to IPA.
